# Barriers and enablers to implementing a virtual tertiary-regional Telemedicine Rounding and Consultation (TRAC) model of inpatient pediatric care using the Theoretical Domains Framework (TDF) approach: a study protocol

**DOI:** 10.1186/s12913-018-3859-2

**Published:** 2019-01-11

**Authors:** Sumedh Bele, Christine Cassidy, Janet Curran, David W. Johnson, Chad Saunders, J.A. Michelle Bailey

**Affiliations:** 1 Department of Community Health Sciences, University of Calgary, Calgary, AB Canada; 2 School of Nursing, Dalhousie University, Halifax, NS Canada; 3 Department of Pediatrics, University of Calgary, Calgary, AB Canada; 4 Haskayne School of Business, University of Calgary, Calgary, AB Canada

**Keywords:** Telemedicine, Ehealth, Pediatric care, Theoretical domains framework

## Abstract

**Background:**

Over-occupancy at the two tertiary pediatric care hospitals in Alberta, Canada is steadily increasing with simultaneous decline in occupancy of pediatric beds at regional hospitals. Over-occupancy negatively impacts timeliness and potentially, the safety of patient care provided at these two tertiary hospitals. In contrast, underutilization of pediatric beds at regional hospitals poses the risk of losing beds provincially, dilution of regional pediatric expertise and potential erosion of confidence by regional providers. One approach to the current situation in provincial pediatric care capacity is development of telemedicine based innovative models of care that increase the population of patients cared for in regional pediatric beds. A Telemedicine Rounding and Consultation (TRAC) model involves discussing patient care or aspects of their care using telemedicine by employing visual displays, audio and information sharing between tertiary and regional hospitals. To facilitate implementation of a TRAC model, it is essential to understand the perceived barriers among its potential users in local context. The current study utilizes qualitative methodologies to assess these perceived clinician barriers to inform a future pilot and evaluation of this innovative virtual pediatric tertiary-regional collaborative care model in Alberta.

**Methods:**

We will use a qualitative descriptive design guided by the Theoretical Domain Framework (TDF) to systematically identify the tertiary and regional clinical stakeholder’s perceived barriers and enablers to the implementation of proposed TRAC model of inpatient pediatric care. Semi-structured interviews and focus groups with pediatricians, nurses and allied health professionals, administrators, and family members will be conducted to identify key barriers and enablers to implementation of the TRAC model using TDF. Appropriate behaviour change techniques will be identified to develop potential intervention strategies to overcome identified barriers. These intervention strategies will facilitate implementation of the TRAC model during the pilot phase.

**Discussion:**

The proposed TRAC model has the potential to address the imbalance between utilization of regional and tertiary inpatient pediatric facilities in Alberta. Knowledge generated regarding barriers and enablers to the TRAC model and the process outlined in this study could be used by health services researchers to develop similar telemedicine-based interventions in Canada and other parts of the world.

**Electronic supplementary material:**

The online version of this article (10.1186/s12913-018-3859-2) contains supplementary material, which is available to authorized users.

## Background

Inpatient pediatric patient care in the province of Alberta is provided through two tertiary pediatric care hospitals, Alberta Children’s Hospital (ACH) and Stollery Children’s Hospital and several regional hospitals. Over-occupancy at the two tertiary pediatric care hospitals is steadily increasing with simultaneous decline in occupancy of pediatric beds at regional hospitals [1]. At ACH, the average occupancy rate in 2015/16 was 95.1% with the site experiencing greater than 100% capacity 197 days of the year against the recommended average bed occupancy of 82–85% [2]. Nineteen percent of Medicine Hat Regional Hospital (MHRH) catchment area pediatric admissions occur at ACH, which represents 28% of total pediatric inpatient days of stay. MHRH pediatric unit’s average bed occupancy in the same time period was 57%. This data highlights the imbalance of regional bed usage between tertiary and regional hospitals. Over-occupancy negatively impacts timeliness and potentially, the safety of patient care and increase the stress on healthcare providers [3–5]. In contrast, underutilization of pediatric beds at regional hospitals poses the risk of losing beds provincially, dilution of regional pediatric expertise and potential erosion of confidence by regional pediatric healthcare providers [6].

One approach to the current situation in provincial pediatric care capacity is development of telemedicine based innovative models of care that increase the population of patients cared for in regional pediatric beds. Telemedicine is defined as “healing at a distance” and broadly means the use of information and communication technologies to overcome geographical barriers, and increase access to health care services [7]. Telemedicine is a tool to increase efficiency, improve access, improve quality of care and facilitate care over distance [8]. Previous studies have shown that telemedicine can help transform health care delivery for pediatric patients by improving accessibility of care among underserved populations with more complex health needs [9]. An integrated telemedicine practice accompanied by enhanced coordination, organization and implementation of health care services can impact positively on the quality of care [10]. Further, a recent study highlighted the complementary role of telemedicine strategies for pediatric subspecialty care and role of telemedicine in enhancing personal relationships between rural pediatricians and subspecialists [11].

**Fig. 1:**
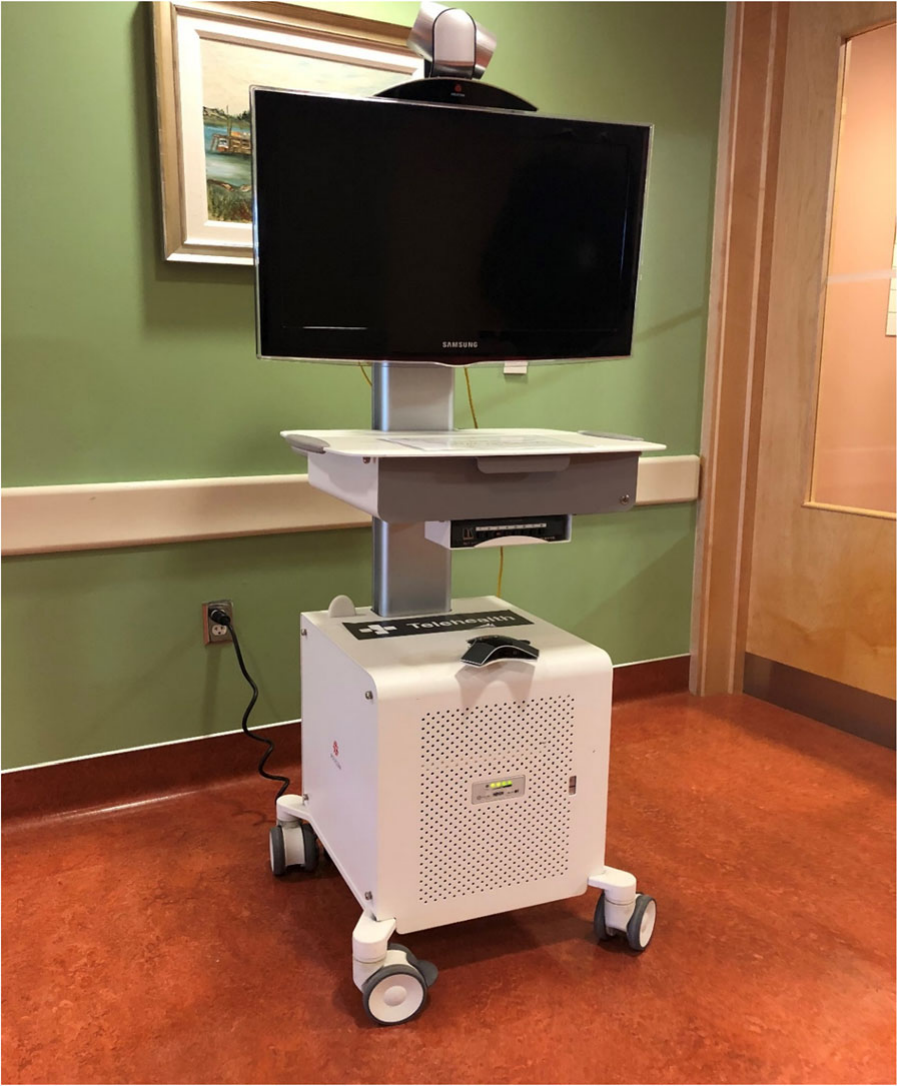
Mobile telemedicine cart capable of real time audio-visual transmission

The proposed innovative virtual care model is a telemedicine facilitated daily rounds and consultations model, abbreviated as the ‘Telemedicine Rounding and Consultation’ (TRAC) model (Table 1). It will involve discussing patient care or aspects of their care using telemedicine cart which employs visual displays, audio and information sharing between tertiary and regional hospitals (Fig. 1). Patients to be included in daily joint rounds are chosen from ACH and/or MHRH inpatient units. Patients to be included in the TRAC model of care are those who may benefit from a collaborative care discussion for some aspect of their care. The term “rounds” in the TRAC model of care refers to a care team discussing a patient care plan. ‘Family Centered Rounds’ may also occur at the patient bedside and with the family present when appropriate. Family presence at rounding is preferred, as it promotes family-centered care where patients and families participate in the discussion including sharing information, asking questions and aiding in decisions about the patient’s care plan [12]. Meeting room rounding can also be used for rounds or education. Rounding will be set up daily, at least from Monday to Friday, with patients included in the daily rounds chosen by care teams at both sites. During the daily rounds time, there will be a brief review of patients at ACH who are from the MHRH area and MHRH patients who may require tertiary services. This allows both teams to be aware of potential patients who may benefit from the TRAC model of care in upcoming days. Post-transfer rounding between tertiary and regional hospitals will be included to ensure the continuum of care for the patients. Additional telemedicine or phone contact will be used to keep the teams updated about the changes in the patient status during the day. ACH and MHRH were chosen as optimal pilot sites based on existing telemedicine champions, ongoing collaboration, and successful piloting of other provincial initiatives at these sites.

**Table 1:** Key features of Telemedicine Rounding and Consultation (TRAC) Model

□ Daily inpatient rounding:	
■ Monday to Friday	
■ Health care teams meet to discuss a patient’s medical care plans.	
■ Bedside rounds (rounds that occur at the bedside with the patient and family included in care planning) when able/appropriate.	
□ Telemedicine facilitated:	
■ Real time audio-visual transmission using telemedicine cart (Fig. 1).	
□ Tertiary-Regional Collaboration:	
■ Collaboration between Alberta Children’s Hospital (tertiary) and Medicine Hat Regional Hospital (regional).	
□ Multidisciplinary teams:	
■ General pediatricians, subspecialists, allied health professionals, nurses, nurse educators (based on patient needs)

Implementation of the TRAC model of inpatient pediatric care will likely require changes in both individual and collective behaviours of healthcare professionals. To refine and facilitate the implementation of TRAC model, it is essential to understand the perceived barriers and enablers to TRAC model among its potential users in local context. This study will use the Theoretical Domains Framework (TDF) to systematically explore perceived clinical barriers and enablers to the implementation of the TRAC model. The TDF is a framework that consists of 84 component constructs which have been refined into 14 theoretical domains [13, 14] (Table 2). The TDF simplifies the complexities associated with behaviour change by providing a theory-informed framework to view the cognitive, affective, social and environmental influences on behaviour [15]. The TDF has successfully been applied to identify barriers and enablers to nurses’ use of electronic medication management systems [16], explore health professionals’ perceived determinants to adherence to multiple evidence-based indicators in primary care [17], and to develop targeted, theory-informed interventions to improve physical therapist management of the risk of falls after discharge [18]. The TDF facilitates the progression from theory-based investigation to intervention design by providing a theoretical basis to understand potential barriers for slow uptake of evidence into practice and ways to overcome these barriers [15]. Relevant and feasible behaviour change techniques (BCTs) to include in an implementation strategy will be identified using the APEASE (affordability, practicability, effectiveness, acceptability, side-effects/safety and equity) criteria [19]. Whilst some evidence exists for the barriers to uptake of telemedicine in different clinical areas [20–22], a comprehensive, systematic, and theory-informed exploration of barriers to telemedicine in pediatric care in Canada is lacking. This study attempts to fill this current knowledge gap by providing insights into the development of telemedicine facilitated model of pediatric care in Canada. The objectives of this study are to: 1. Systematically explore barriers and enablers to adopting the TRAC model of inpatient pediatric care; 2. Identify BCTs to successfully address these barriers; 3. Develop an implementation strategy to overcome the barriers and enhance the facilitators to implementing the TRAC model of care into practice. This paper describes the proposed process of exploring barriers and enablers to TRAC model using TDF approach. The current study is part of a larger project, sponsored by the Maternal, Newborn, Child & Youth Strategic Clinical Network (MNCY SCN™^)^, which aims to develop an innovative virtual pediatric care model, pilot, and evaluate the model at ACH and MHRH and if the pilot is successful, expand it throughout the province of Alberta.

**Table 2:** The Theoretical Domains Framework with definitions

Domain	Definition
1. Knowledge	An awareness of the existence of something
2. Skills	An ability or proficiency acquired through practice
3. Social/professional role & identity	A coherent set of behaviours and displayed personal qualities of an individual in a social or work setting
4. Beliefs about capabilities	Acceptance of the truth, reality, or validity about an ability, talent, or facility that a person can put to constructive use
5. Optimism	The confidence that things will happen for the best or that desired goals will be attained
6. Beliefs about consequences	Acceptance of the truth, reality, or validity about outcomes of a behaviour in a given situation
7. Reinforcement	Increasing the probability of a response by arranging a dependent relationship, or contingency, between the response and a given stimulus
8. Intentions	A conscious decision to perform a behaviour or a resolve to act in a certain way
9. Goals	Mental representations of outcomes or end states that an individual wants to achieve
10. Memory, attention & decision processes	The ability to retain information, focus selectively on aspects of the environment and choose between two or more alternatives
11. Environmental context & resources	Any circumstance of a person’s situation or environment that discourages or encourages the development of skills and abilities, independence, social competence, and adaptive behaviour
12. Social influences	Those interpersonal processes that can cause individuals to change their thoughts, feelings, or behaviours
13. Emotion	A complex reaction pattern, involving experiential, behavioural, and physiological elements, by which the individual attempts to deal with a personally significant matter or event
14. Behavioural regulation	Anything aimed at managing or changing objectively observed or measured actions

[From: Cane, J., D. O’Connor, and S. Michie, Validation of the theoretical domains framework for use in behaviour change and implementation research. Implement Sci, 2012. 7: p. 37]

## Methods

### Study design

**Fig. 2:**
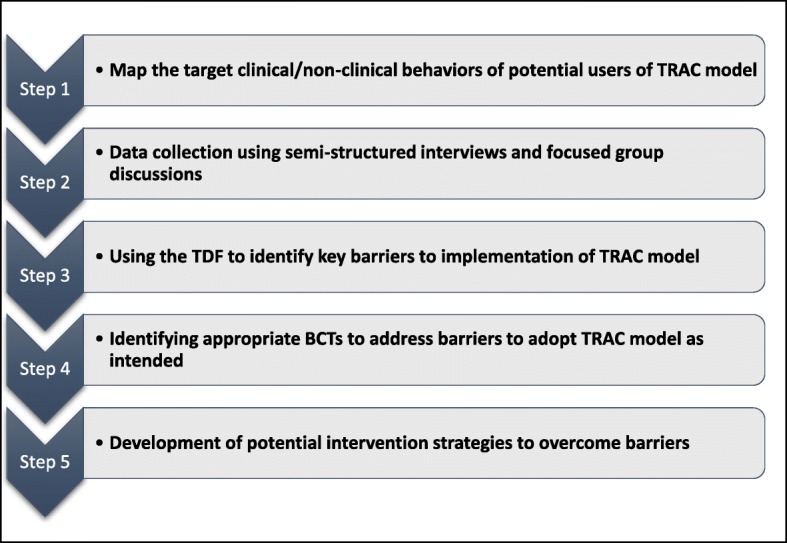
Study design flowchart using the Theoretical Domains Framework (TDF) approach from

A qualitative descriptive design [23] will be used to systematically explore tertiary and regional clinical stakeholders’ perceived barriers and enablers to the implementation of the proposed TRAC model of inpatient pediatric care in Alberta (Fig. 2). The goal of qualitative descriptive design is to determine the meaning of a phenomenon through description. It also aids in developing and exploring the concepts by offering the flexibility to understand the phenomenon in the context of study setting [24].

### Study setting

ACH, located in the city of Calgary is the largest tertiary level public pediatric hospital in Alberta with 141 beds. MHRH is a medical facility located in Medicine Hat city, 300 km southeast of Calgary, serving a catchment area of 117,000 residents. MHRH has 325 beds in total including 10 beds for pediatric patients (outside of the neonatal intensive care unit).

### Objectives of the study

#### Objective 1: Identify barriers and enablers

We will conduct 14 semi-structured individual interviews and one focus group at ACH and 12 semi-structured individual interviews and one focus group at MHRH to better understand participants’ perceived barriers and enablers to implementing the TRAC model into practice.

#### Recruitment of participants

We will use a stratified purposive sampling strategy with convenience sampling techniques to recruit a diverse sample of tertiary and regional clinical stakeholders including pediatricians, nurses and allied health professionals, administrators, and family members or care takers of the pediatric patients. We aim to recruit 42 participants in total (Tables 3 and 4). Individual interviews will be conducted at both ACH and MHRH with health care providers, administrators, and family members. Health care providers including general pediatricians, subspecialists, nurses and allied care providers who would be the potential users of TRAC model of care would be selected at both the sites. Administrative leaders with oversight of pediatric inpatient programs will be recruited from both sites. Family members recruited for the study will be family members or care givers of pediatric patients from MHRH catchment area who have received inpatient pediatric care at ACH or MHRH in the last three years and family members of patients who have received care at ACH in the last 3 years. The ACH Family Centered Care committee and pediatricians providing care at MHRH will assist in identifying potential participants. Family members will be included in the study as the representatives for patients and families who could potentially benefit from TRAC model of care.. In addition to individual interviews, focus groups will be conducted at ACH and MHRH. As part of the focused groups, frontline clinicians and allied health professionals would be invited to participate in interviewer guided small-group discussion with fellow health care providers with similar background or experiences with providing pediatric care. Frontline staff is chosen for focus groups because they would be the primary agents for implementing TRAC model. Frontline staff provide care as a group, so focus group setting will elicit their perceived barriers and enablers to TRAC model in real-life setting by responding and building on each other’s responses. To mitigate the potential influence of pre-existing professional relationships and power differential, administrators will not be included in any of the focus groups.

**Table 3:** Sample of total potential participants for semi-structured interviews and focused group discussions

Participant’s current role	Alberta Children’s Hospital (ACH)	Medicine Hat Regional Hospital (MHRH)	Total
Pediatricians	6	4	10
Nurses and allied health professionals	9	12	21
Administrators	3	3	6
Families	2	3	5
Total	20*	22*	42*

*note: additional interviews may be required to reach saturation of identified themes

**Table 4:** Sub-sample of potential participants for focused group discussions at ACH and MHRH

Participant’s current role	Alberta Children’s Hospital (ACH)	Medicine Hat Regional Hospital (MHRH)	Total
Pediatricians	1	1	2
Nurses and allied health professionals	4	5	9
Total	5	6	11

Inclusion of health providers along with family members both at ACH and MHRH will promote integration of knowledge for users through our integrated knowledge translation strategy. We will offer each participant a $15 gift card honorarium for their participation.

### Materials

The interview guide includes two to four questions for each of the 14 domains of the TDF (see Additional file 1). Before each interview or focus group, interviewers will explain the TRAC model of inpatient pediatric care in more detail. The interview guide aims to elicit current knowledge, perceptions and explore the barriers and enablers to adopting the TRAC model of inpatient pediatric care. Each interview and focus group will be audio recorded and last approximately 45–60 min.

### Procedure

Potential allied health professional and administrative participants at each study site will be invited by the primary investigator for one-on-one interviews or focus groups at ACH or MHRH. In compliance with the subject recruitment practice of Alberta Health Services, primary investigator will ask the manager of the clinical area(s) to send out an invitation to the staff on behalf of the researcher. The researchers will make it clear that participation is voluntary and will not affect their employment.

Participants will be required to sign a written consent form to indicate their willingness to participate in this study. Only anonymized quotes will be published. Additional written consent will be obtained to audio record the interviews and focus groups. Team members with experience in conducting qualitative research and its application using the TDF approach developed the interview guides for the semi-structured interviews and focus groups. All the interviews and focus groups will be facilitated by one or two interviewers with experience in qualitative data collection. Each health provider will complete a brief form to gather demographic information on their care provider group (pediatrician, pediatric subspecialist, nurse, other allied health, or administrator) and their work location (ACH or MHRH). The family members will complete a separate form to gather information about their age, area of residence, level of education, employment status, age of their child/children and frequency of care received at ACH and/or MHRH for their child/children. Additional interviews will be conducted until data saturation is reached. Data is considered saturated when we reach a point in our analysis where gathering more data will not provide us any new information related to our research question.

### Data analysis

Interview and focus group recordings will be transcribed verbatim. A qualitative data analysis software NVivo 11 (QSR, Melbourne, Australia) will be used to code, organize, and manage the data to facilitate data interpretations. Data will be analyzed using a directed content analysis approach (18). Content analysis is a systematic coding and categorization approach to qualitative data analysis used to examine trends and patterns of the data, and determine the frequency and relationships of the words used by participants (18). To test the agreement on coding for each domain of the TDF, two randomly selected transcripts will be coded independently by two different members of the research team with qualitative research experience. Discrepancies will be resolved through discussion. All the remaining transcripts will be coded by a single reviewer.

For the initial analysis, the reviewers will read the transcripts and categorize similar statements into the 14 TDF domains. Second, an inductive coding approach [25] will be used to generate subcategories of specific beliefs of participants within the initial coding scheme of the 14 TDF domains. A specific belief is a group of similar responses that suggest the belief may influence the target behaviour (13). Lastly, the coded data will be further inductively examined to identify relevant theoretical domains to the target behaviour (telemedicine-based daily rounds). We will examine trends, patterns, frequencies, and relationships of the words used by the participants to determine: 1. Any conflicting beliefs within a domain; 2. The frequency of specific beliefs across the data; and 3. The likely strength of the impact of a belief on the behaviour. This approach will allow for the inclusion of barriers and enablers under both predefined TDF domains and emergent codes. Irrelevant domains will be excluded from further analysis. Sub-analysis of the data to identify perception of the barriers and enablers will be conducted based on the study site (ACH vs MHRH). Thematic content will be assessed to tabulate barriers and enablers for proposed TRAC model of inpatient pediatric care within each TDF domains.

### Objective 2: Identify BCTs

After identifying the barriers and enablers in step 2, we will use the BCT Taxonomy version 1 [26], a grouping of 93 hierarchically clustered techniques, to identify potential BCTs known to be effective at overcoming the identified barriers and enhancing the enablers to implementing the TRAC model. It is a standardized language for describing the ‘active ingredients’ in interventions. Michie and colleagues developed a matrix which maps relevant BCTs to corresponding TDF domains [26]. Research team members with expertise in pediatric care at these two study sites and in intervention design will map the identified barriers and enablers from Objective 1 to BCTs that are likely to change behaviour [19].

### Objective 3: Development of potential intervention strategies to overcome barriers

The project team will use the findings from Objectives 1 and 2 to develop a final implementation strategy for piloting TRAC model at the two sites. Through discussion, the research team will consult with potential users of the TRAC model and apply the APEASE criteria to the relevant BCTs, potential intervention content and mode of delivery to explore its appropriateness and feasibility for the local context. The APEASE criteria (affordability, practicability, effectiveness and cost-effectiveness, acceptability, safety, and equity) are used to guide decision-making during intervention design.

## Discussion

Health information technology has been used to facilitate care of pediatric patients with a range of diseases requiring follow-up and involving participation of both a caregiver and health care providers to improve continuity of care [27]. Telemedicine has the potential to improve quality of care and enhance accessibility by bridging geographical barriers to provide care over the distance [8]. Despite emergence of telemedicine as a feasible and efficient option for pediatric patients, providers, and payers that results in high-quality, cost-effective care, there is a paucity of telemedicine implementation literature. [28, 29].Evidence from Australia suggest that telemedicine can be effective in linking specialized pediatric care with home care, save the cost of delivering pediatric health services and clinicians have positive perception of telemedicine. However, this research groups have also highlighted the need to understand perceived usefulness of telemedicine by some clinicians and to gather further evidence to support its use in paediatric acute care [30–32].

Like in adult care, telemedicine technologies in pediatric care are used to deliver the same or enhanced care that is currently offered, but various factors add an additional layer of complexities while delivering telemedicine facilitated care for pediatric patients [33]. In pediatric care, questions from the medical team are usually directed at patient’s parents/caregivers, so acceptability of such technology by both the patient and their family caregivers is the key for delivering pediatric care through telemedicine.

Hospitalization impacts pediatric patient’s schooling and ultimately psychosocial development [34], so the outcomes like reduction in length of stay due to delivering telemedicine facilitated care in pediatric population might differ from the adult population. Parents/caregivers are considered primary agents involved in the delivery of pediatric health care. Adverse events like hospitalization and travelling associated with pediatric patient’s health conditions put family members under high levels of stress and anxiety. Parents’ poor psychosocial functioning may deeply influence the child’s adherence to the care and impact of the disease [35]. While the literature on developing and implementation of telemedicine in adult care is widely available, and the impact of such technologies are extensively studied, the delivery of care and outcomes of interest vary between adult and pediatric health care services, further highlighting the need for telemedicine research that is specific to children.

Telemedicine’s potential to transform the health care system could not be fully achieved without understanding the perceived barriers and enablers for its uptake by the stakeholders of clinical care. This study will contribute to the understanding of the telemedicine facilitated models of care implementation barriers and enablers.

The findings from this study will be used to guide the development and implementation process of TRAC model during the pilot phase. The evidence generated from this study can also be used to guide further programmatic research efforts to scale-up the use of telemedicine across geographies. The overarching goal of this pilot evaluation is to determine if the TRAC model of inpatient pediatric care increases the use of regional pediatric beds. The proposed TRAC model provides an opportunity for health providers to maintain continuity of care and address the imbalance between utilization of regional and tertiary inpatient pediatric facilities in Alberta. The use of telemedicine is associated with reduction in travel and healthcare costs [36]. This patient-centered care approach will benefit the patients from regional catchment areas who will access tertiary hospital level care expertise and resources within their own region without travelling to the tertiary level hospital. Families will also benefit by saving travel time, minimizing time away from work and being able to stay better connected to their community support networks.

The TRAC model of pediatric inpatient care may be expandable in scope and scale provincially if the pilot study determines this model to be feasible and acceptable to health care providers and families. The province-wide application of TRAC model would increase regional pediatric bed usage in multiple regional sites thereby positively impacting the current tertiary over-occupancy crisis in Alberta. In addition to addressing the imbalance in utilization of pediatric capacities between regional and tertiary hospitals, it is anticipated that this project will enhance tertiary-regional collaboration thereby supporting numerous other provincial goals such as provincial pediatric guideline implementation and pediatric workforce sustainability [37]. MNCY SCN™ is uniquely positioned as the premier vehicle for engaging front-line clinicians, researchers, and partners to drive sustainable improvements and innovations across the continuum of maternal and child care in Alberta. Therefore, the findings of this study will be disseminated as pediatric health services research-generated knowledge through MNCY SCN™. The results of this study will also be shared with the study partners at the ACH and MHRH and participants will receive an executive summary of the findings. Dissemination will also occur through presentations and peer-reviewed publications.

Two major strengths of this study lie in its use of a systematic approach to explore the barriers and enablers to the proposed TRAC model using the TDF and its inclusion of range of health professionals and family members as potential users of the TRAC model. TDF has already been used in range of research contexts including telemedicine interventions [38], so this study will contribute to the growing TDF and intervention design literature. However, several limitations need to be acknowledged. Firstly, the TDF provides a framework to elicit barriers pertaining to each domain but it does not specify relationships between the domains [39]. Second, focus groups allow for gathering relevant information in a short period of time and offer insights on the existing contradictions and differences in the individual opinions of the participants; however, pre-existing professional relationships and power differential may influence group dynamics during the focus groups. This limitation would be potentially neutralized by excluding administrators from the focus groups.

## Conclusion

By identifying the barriers and enablers to a virtual telemedicine facilitated model within Alberta’s health system, we expect to gain better insights into stakeholders’ perceptions of telemedicine facilitated care in the province. Although the results of this study might not be directly generalizable to other health care settings, the knowledge generated regarding barriers and enablers to the TRAC model and the process outlined in this study could be used by the health services researchers to develop similar telemedicine-based interventions in Canada or other parts of the world.

## Additional file

Additional file 1: **Table S1.** Interview guide for health care providers. **Table S2.** Interview guide for family members. **Table S3.** Basic information form for healthcare providers. **Table S4.** Basic information form for family members. (DOCX 28 kb)

## Abbreviations

ACH: Alberta Children’s Hospital
AHS: Alberta Health Services
BCTs: Behaviour change techniques
MHRH: Medicine Hat Regional Hospital
MNCY SCN™: The Maternal, Newborn, Child & Youth Strategic Clinical Network
TDF: Theoretical Domains Framework
TRAC model: Telemedicine Rounding and Consultation model
