# Supplementary material for: Barriers and enablers to implementing a virtual tertiary-regional Telemedicine Rounding and Consultation (TRAC) model of inpatient pediatric care using the Theoretical Domains Framework (TDF) approach: a study protocol
Source: BMC Health Serv Res. 2019 Jan 11;19:29. doi: 10.1186/s12913-018-3859-2 (PMC6330415; doi:10.1186/s12913-018-3859-2)
Supplement: Supplementary file 1 — Table S1. Interview guide for health care providers. Table S2. Interview guide for family members. Table S3. Basic information form for healthcare providers. Table S4. Basic information form for family members. (DOCX 28 kb) [file 12913_2018_3859_MOESM1_ESM.docx]

**Appendices**

**Appendix 1: Interview guide for health care providers**

| **Theoretical Domain** | **Definition (Cane et al. 2012)** | **Sample Question** |
| --- | --- | --- |
| **Knowledge** | An awareness of the existence of something | 1. Have you ever heard about the use of telemedicine/ehealth to provide care at a distance?  2. Are you familiar with using any telehealth/ehealth technologies to provide care at a distance?  3. Are you familiar with the use of telemedicine for daily care rounds and consultations? |
| **Skills** | An ability or proficiency acquired through practice | 1. In your opinion, what skills would be needed to conduct telemedicine facilitated daily care rounds and consultations? (prompt –are there any other skills that you need?) |
| **Social/Professional Role & Identity** | A coherent set of behaviors and displayed personal qualities of an individual in a social or work setting | 1.What do you think would be the purpose of telehealth facilitated daily rounds and consultations between MHRH and ACH?  2. Is providing care through telehealth based daily rounds and consultations encouraged or discouraged by your professional association?  3. Is there anything about your professional role as _______, that would influence how you provide telehealth facilitated daily care rounds and consultations? |
| **Beliefs about Capabilities** | Acceptance of the truth, reality, or validity about an ability, talent or facility that a person can put to constructive use | 1. How easy or difficult would it be to provide telemedicine facilitated daily care rounds and consultations? (prompt – what would make it easy or difficult for you?)  2. What challenges do you foresee encountering by providing telemedicine facilitated daily care rounds and consultations?  3. How confident do you feel in your ability to provide telemedicine facilitated daily care rounds and consultations? |
| **Optimism** | The confidence that things will happen for the best or that desired goals will be attained | 1.How confident are you that patients will receive good care through telemedicine facilitated daily care rounds and consultations? |
| **Beliefs about Consequences** | Acceptance of the truth, reality or validity about outcomes of a behavior in a given situation | 1. What do you think are the consequences of providing telemedicine facilitated daily care rounds and consultations between MHRH and ACH?  2. What do you think are the benefits of providing telemedicine facilitated daily care rounds and consultations between MHRH and ACH?  3. Are there any harms in providing telemedicine facilitated daily care rounds and consultations for health providers?  4. What do you think is the balance of potential benefits and potential harms for providing telemedicine facilitated daily care rounds and consultations between MHRH and ACH? Does one outweigh the other? |
| **Reinforcement** | Increasing the probability of a response by arranging a dependent relationship, contingency, between the response and a given stimulus | 1. Are there any incentives for you to provide telemedicine facilitated daily care rounds and consultations? If yes, what are they?  2. What do you think will happen if this telemedicine facilitated daily care rounds and consultations are incorporated in your practice?  3. What do you think will happen if this telemedicine facilitated daily care rounds and consultations are NOT incorporated in your practice? |
| **Intentions** | A conscious decision to perform a behaviour or a resolve to act in a certain way | 1. On a scale of 1-10 and 10 being very important, how important do you think it is for you personally to provide telemedicine facilitated daily care rounds and consultations? Why? |
| **Goals** | Mental representations of outcomes or end states that an individual wants to achieve | 1. Would the goal of providing care to patients using telemedicine facilitated daily care rounds and consultations be compatible with your usual practice? (prompt – why?) |
| **Memory, Attention & Decision Processes** | The ability to retain information, focus selectively on aspects of the environment and choose between two or more alternatives | 1. Are there situations when you think it would be difficult to provide care to patients using telemedicine facilitated daily care rounds and consultations (prompt – can you tell me what is it about these situations that make it difficult)  2. What would help to overcome such situations? |
| **Environmental Context & Resources** | Any circumstance of a person’s situation or environment that discourages or encourages the development of skills and abilities, independence, social competence, and adaptive behavior | 1.What factors in your clinical environment would influence your decision to provide telemedicine facilitated daily care rounds and consultations? (prompt – to what extent do you feel these factors influence your ability)  2. What factors outside of your clinical environment would influence the use of telemedicine facilitated daily care rounds and consultations?  3. Are there competing tasks or time constraints that would influence your decision to provide telemedicine facilitated daily care rounds and consultations? |
| **Social Influences** | Those interpersonal processes that can cause individuals to change their thoughts, feelings, or behaviors | 1. Have you ever discussed the potential of providing telemedicine facilitated daily care rounds and consultations with other physicians or nurses in your care team? (Prompt- What was the summary of the discussion)  2. Would clinicians or any other member in your care team influence the use of telemedicine facilitated daily care rounds and consultations? (prompt - How would they influence? To what extent?) |
| **Emotion** | A complex reaction pattern, involving experiential, behavioural, and physiological elements, by which the individual attempts to deal with a personally significant matter or event | 1. Does the idea of having telemedicine facilitated daily care rounds and consultations evoke an emotional response in you? (prompt – would you feel worried or concerned about it?)  2. Would your patient’s emotions/preferences ever affect your decision of providing telemedicine facilitated daily care rounds and consultations? (prompt – what about patient’s families, would their emotions/preferences ever affect the decision of using telemedicine facilitated daily care rounds and consultations?) |
| **Behavioural Regulation** | Anything aimed at managing or changing objectively observed or measured actions | 1. What do you think is needed to ensure that we successfully implement telemedicine facilitated daily care rounds and consultations?  (prompt – things specific to you or your unit or your hospital) |

**Appendix 2: Interview guide for family members**

| **Theoretical Domain** | **Definition (Cane et al. 2012)** | **Sample Question** |
| --- | --- | --- |
| **Knowledge** | An awareness of the existence of something | 1. Have you ever heard about the use of telemedicine/ehealth to provide/receive care at a distance?  2. Are you familiar with using any telehealth/ehealth technologies to receive care at a distance?  3. Are you familiar with the use of telemedicine for daily care rounds and consultations? |
| **Skills** | An ability or proficiency acquired through practice | 1. In your opinion, what skills would be needed to participate in telemedicine facilitated daily care rounds and consultations? (prompt –are there any other skills that you need?) |
| **Social/Professional Role & Identity** | A coherent set of behaviors and displayed personal qualities of an individual in a social or work setting | 1.What do you think would be the purpose of telehealth facilitated daily rounds and consultations between MHRH and ACH?  2. Is there anything about your role as a family member, that would influence how your child receive telehealth facilitated daily care rounds and consultations? |
| **Beliefs about Capabilities** | Acceptance of the truth, reality, or validity about an ability, talent or facility that a person can put to constructive use | 1. How easy or difficult would it be to participate in telemedicine facilitated daily care rounds and consultations? (prompt – what would make it easy or difficult for you?)  2. What challenges do you foresee encountering by receiving your child’s care through telemedicine facilitated daily care rounds and consultations? |
| **Optimism** | The confidence that things will happen for the best or that desired goals will be attained | 1.How confident are you that your child will receive good care through telemedicine facilitated daily care rounds and consultations? |
| **Beliefs about Consequences** | Acceptance of the truth, reality or validity about outcomes of a behavior in a given situation | 1a. (for MHRH area residents) What do you think are the consequences for hospitalized children from your regional area receiving telemedicine facilitated daily care rounds and consultations between MHRH and ACH?  1b. (for non MHRH area residents) What do you think are the consequences for hospitalized children from a regional area like the Medicine Hat Regional Hospital receiving telemedicine facilitated daily care rounds and consultations between MHRH and ACH?  2a. (for MHRH area residents) What do you think are the benefits for hospitalized children you’re your regional area having access to telemedicine facilitated daily care rounds and consultations between MHRH and ACH?  2b. (for non MHRH area residents) What do you think are the benefits for hospitalized children in a regional area having access to telemedicine facilitated daily care rounds and consultations between the regional site and ACH?  3. Are there any harms for hospitalized children receiving telemedicine facilitated daily care rounds and consultations?  4. What do you think is the balance of potential benefits and potential harms of receiving telemedicine facilitated daily care rounds and consultations between MHRH and ACH? Does one outweigh the other? |
| **Reinforcement** | Increasing the probability of a response by arranging a dependent relationship, contingency, between the response and a given stimulus | 1. Are there any incentives for you to participate in telemedicine facilitated daily care rounds and consultations? If yes, what are they?  2. What do you think will happen if this telemedicine facilitated daily care rounds and consultations are incorporated into the inpatient care for patients from the Medicine Hat area?  3. What do you think will happen if this telemedicine facilitated daily care rounds and consultations are NOT incorporated into the inpatient care for patients from the Medicine Hat area? |
| **Intentions** | A conscious decision to perform a behaviour or a resolve to act in a certain way | 1. On a scale of 1-10 and 10 being very important, how important do you think it is for you if your child was admitted at ACH/MHCH, to participate in telemedicine facilitated daily care rounds and consultations? Why? |
| **Goals** | Mental representations of outcomes or end states that an individual wants to achieve | 1. Would the goal of receiving care for your child using telemedicine facilitated daily care rounds and consultations be compatible with your expectations? (prompt – why?) |
| **Memory, Attention & Decision Processes** | The ability to retain information, focus selectively on aspects of the environment and choose between two or more alternatives | 1. Are there situations when you think it would be difficult to receive care using telemedicine facilitated daily care rounds and consultations (prompt – can you tell me what it is about these situations that make it difficult)  2. What would help to overcome such situations? |
| **Environmental Context & Resources** | Any circumstance of a person’s situation or environment that discourages or encourages the development of skills and abilities, independence, social competence, and adaptive behavior | 1.What factors in your clinical environment would influence your decision to participate in telemedicine facilitated daily care rounds and consultations? (prompt – to what extent do you feel these factors influence your ability)  2. What factors outside of your clinical environment would influence the use of telemedicine facilitated daily care rounds and consultations?  3. Are there competing tasks or time constraints that would influence your decision to receive telemedicine facilitated daily care rounds and consultations? |
| **Social Influences** | Those interpersonal processes that can cause individuals to change their thoughts, feelings, or behaviors | 1. Have you ever discussed the potential of receiving telemedicine facilitated daily care rounds and consultations with your physicians or nurses? (Prompt= What was the summary of the discussion)  2. Would clinicians or any other member in your child’s care team influence your thoughts on use of telemedicine facilitated daily care rounds and consultations? (prompt - How would they influence? To what extent?) |
| **Emotion** | A complex reaction pattern, involving experiential, behavioural, and physiological elements, by which the individual attempts to deal with a personally significant matter or event | 1. Does the idea of having telemedicine facilitated daily care rounds and consultations evoke an emotional response in you? (prompt – would you feel worried or concerned about it?)  2. Would your care providers/ pediatrician’s emotions/preferences ever affect your decision of receiving telemedicine facilitated daily care rounds and consultations? (prompt – what about other family members? would their emotions/preferences ever affect the decision of receiving telemedicine facilitated daily care rounds and consultations?) |
| **Behavioural Regulation** | Anything aimed at managing or changing objectively observed or measured actions | 1. What do you think is needed to ensure that we successfully implement telemedicine facilitated daily care rounds and consultations?  (prompt – things specific to your child’s care) |

**Appendix 3: Basic information form for healthcare providers**

1. Study participant number - _____________ (to be filled by interviewer)
2. Current place of work

- Alberta Children’s Hospital
- Medicine Hat Regional Hospital

1. Current role as a healthcare provider

- General pediatrician
- Specialist pediatrician
- Nurse
- Allied health professional
- Administrator

**Appendix 4: Basic information form for family members**

1. Study participant number - _____________ (to be filled by interviewer)
2. Role as family member

- Father
- Mother
- Grandfather
- Grandmother
- Other (please specify) _________________

1. Current place of residence

- Calgary region
- Medicine Hat region

1. Age of the participant

- Under 18 years
- 18 to 24 years
- 25 to 34 years
- 35 to 44 years
- 45 to 54 years
- 55 to 64 years
- Age 65 or older

1. Level of education

- Haven’t completed high school
- High school graduate
- Completed some college
- Associate degree/diploma
- Bachelor's degree
- Completed some postgraduate
- Master's degree
- Ph.D., law or medical degree
- Other advanced degree beyond a Master's degree
- Other (please specify) ____________________________

1. Current employment status

- Unemployed/ homemaker
- Employed
- Full-time
- Part-time
- Self-employed
- Retired
- Other (please specify) _____________________________

1. Current age of your child/children (can select more than one)

- Neonate/infant (under 1-year old)
- Toddler (1 -2 years old)
- Early childhood (2-5 years old)
- Middle childhood (6-11 years old)
- Early adolescence (12-18 years old)
- Late adolescence (18-21 years old)

1. Number of hospitalizations at Alberta Children’s Hospital for your child/ children in the last one year

- 1
- 2
- 3 or more (if remember, please specify) ________

1. Number of hospitalization at Alberta Children’s Hospital since birth for your child/ children

- 1
- 2-4
- 3-9
- 10 or more (if remember, please specify) __________

1. Number of hospitalizations at Medicine Hat Regional Hospital for your child/ children in the last one year

- 1
- 2
- 3 or more (if remember, please specify) ________

1. Number of hospitalization at Medicine Hat Regional Hospital since birth for your child/ children

- 1
- 2-4
- 3-9
- 10 or more (if remember, please specify) __________
